# The effect of day-neutral mutations in barley and wheat on the interaction between photoperiod and vernalization

**DOI:** 10.1007/s00122-013-2133-6

**Published:** 2013-06-05

**Authors:** Adrian S. Turner, Sébastien Faure, Yang Zhang, David A. Laurie

**Affiliations:** 1 Department of Crop Genetics, John Innes Centre, Colney Lane, Norwich, NR47UH UK; 2 Department of Metabolic Biology, John Innes Centre, Colney Lane, Norwich, NR47UH UK; 3 Present Address: Biogemma, Cereals Genetics and Genomics, 63028 Clermont Ferrand Cedex 2, France

## Abstract

*Vernalization*-*2* (*Vrn*-*2*) is the major flowering repressor in temperate cereals. It is only expressed under long days in wild-type plants. We used two day-neutral (photoperiod insensitive) mutations that allow rapid flowering in short or long days to investigate the day length control of *Vrn*-*2*. The barley (*Hordeum vulgare*) *early maturity8* (*eam8*) mutation affects the barley *ELF3* gene. *eam8* mutants disrupt the circadian clock resulting in elevated expression of *Ppd*-*H1* and the floral activator *HvFT1* under short or long days. When *eam8* was crossed into a genetic background with a vernalization requirement *Vrn*-*2* was expressed under all photoperiods and the early flowering phenotype was partially repressed in unvernalized (UV) plants, likely due to competition between the constitutively active photoperiod pathway and the repressing effect of *Vrn*-*2.* We also investigated the wheat (*Triticum aestivum*) *Ppd*-*D1a* mutation. This differs from *eam8* in causing elevated levels of *Ppd*-*1* and *TaFT1* expression without affecting the circadian clock. We used genotypes that differed in “short-day vernalization”. Short days were effective in promoting flowering in individuals wild type at *Ppd*-*D1*, but not in individuals that carry the *Ppd*-*D1a* mutation. The latter showed *Vrn*-*2* expression in short days. In summary, *eam8* and *Ppd*-*D1a* mimic long days in terms of photoperiod response, causing *Vrn*-*2* to become aberrantly expressed (in short days). As *Ppd*-*D1a* does not affect the circadian clock, this also shows that clock regulation of *Vrn*-*2* operates indirectly through one or more downstream genes, one of which may be *Ppd*-*1*.

**Electronic supplementary material:**

The online version of this article (doi:10.1007/s00122-013-2133-6) contains supplementary material, which is available to authorized users.

## Introduction

The environmental cues of day length (photoperiod) and extended periods of low temperature (vernalization) are used by many plant species to regulate the timing of flowering during the year. Correct timing is an important component of adaptation in crops and has a strong effect on yield (reviewed by Cockram et al. 2007). In the model plant *Arabidopsis thaliana* (Arabidopsis subsequently) the photoperiod pathway involves circadian regulation of *CONSTANS* (*CO*) so that expression peaks approximately 16 h after dawn. Under long-day (LD) conditions the coincidence of light with CO protein allows the induction of *FLOWERING LOCUS T* (*FT*) in vascular tissue in leaves (Valverde et al. 2004). FT protein moves via the phloem to the apex where it interacts with the FD protein to activate *SOC1* and *AP1*, leading to floral transition (Abe et al. 2005; Wigge et al. 2005). The principal flowering repressor in Arabidopsis is the MADS-box transcription factor *FLOWERING LOCUS C* (*FLC*) that represses *FT*, *FD* and *SOC1* (reviewed by Higgins et al. 2010). *FLC* is stably down regulated by epigenetic modification during vernalization (reviewed by Amasino 2010; Crevillén and Dean 2010) allowing inductive pathways, such as photoperiod, to promote flowering.

Well conserved homologues of the core components of the circadian clock and the photoperiod pathway (*GI*, *CO* and *FT*) are found in other plants including cereals, suggesting that the core photoperiod pathway is conserved (reviewed by de Montaigu et al. 2010; Song et al. 2010; Higgins et al. 2010). However, additional genes have been recruited in different lineages. In cereals, examples of the latter are *ID1*, *Ehd7* and *Ghd7* in rice (*Oryza sativa*) (reviewed by Higgins et al. 2010). In temperate cereals such as barley (*H. vulgare*) and bread or pasta wheat (*T. aestivum*, *T. durum,* respectively) the pseudo-response regulator *Ppd*-*1*, which has homology to circadian clock genes of Arabidopsis, is an important regulator of flowering (Turner et al. 2005; Beales et al. 2007; Wilhelm et al. 2009; Shaw et al. 2012). *FT1*, the likely orthologue of *FT* in Arabidopsis, can be regulated by *Ppd*-*1* independently of *CO* (Campoli et al. 2012; Shaw et al. 2012).

In contrast to photoperiod, vernalization pathways show much greater divergence (reviewed by Distelfeld et al. 2009a; Greenup et al. 2009). No homologue of the Arabidopsis *FLC* gene has been identified in cereals. Instead the role of flowering repressor is taken by *VERNALIZATION*-*2* (*Vrn*-*2*), a member of a grass-specific subgroup of CCT domain genes, a family that also includes *CO* and *Ppd*-*1* (Yan et al. 2004; Mizuno and Nakamichi 2005; Higgins et al. 2010). Loss of function mutations of *Vrn*-*2* allow flowering without vernalization (Yan et al. 2004; Distelfeld et al. 2009b).

Two other genes involved in vernalization have been identified in cereals. *Vrn*-*1* is a MADS-box transcription factor related to the *AP1*/*FUL* subgroup in Arabidopsis (Yan et al. 2003; reviewed by Higgins et al. 2010). *Vrn*-*1* expression increases gradually during vernalization (Danyluk et al. 2003; Murai et al. 2003; Yan et al. 2003). *Vrn*-*3* has proved to be the same gene as *FT1* (Yan et al. 2006; reviewed by Higgins et al. 2010) and is called *FT1* in this paper. Winter cereals, which require vernalization, have wild-type alleles at all three loci. Spring cereals, which have no, or reduced, vernalization requirement, have a mutation of one or more of the *Vrn* genes.

Current models for temperate cereals are that VRN-2 protein represses *FT1* (*VRN*-*3*) to prevent flowering, as proposed by Yan et al. (2006). Hemming et al. (2008) demonstrated that overexpression of *HvVrn2* down-regulates *HvFT1* and represses flowering. *Vrn*-*1* is induced during vernalization and represses *Vrn*-*2*, allowing *FT1* expression to be induced by LDs (Trevaskis et al. 2006; Hemming et al. 2008; Distelfeld et al. 2009b; Chen and Dubcovsky 2012). Several lines of evidence have indicated that photoperiod, and photoperiod sensitivity, play a role in the activity of *Vrn*-*2*. Karsai et al. (2005) found that the presence/absence of *HvVrn2* only affected vernalization response in LD; there was no response in SD. In addition, Hemming et al. (2008) found that rapid flowering of plants carrying deletions at *HvVrn2* was dependent on the presence of an active Ppd-H1 allele.

*Vrn*-*2* is expressed principally in leaves of wheat and barley (Yan et al. 2004; Sasani et al. 2009) under LD but not short-day (SD) conditions (Dubcovsky et al. 2006; Trevaskis et al. 2006). This contrasts with Arabidopsis where the expression of FLC is not dependent on day length (Sung et al., 2006). These findings suggest that *Vrn*-*2* acts as a LD repressor, preventing flowering in winter cereals germinating in summer or autumn when day lengths would be sufficient to activate photoperiod response. This is similar to the LD repression role of the related CCT domain gene *Ghd7* in rice (Xue et al. 2008).

Wild-type barley and wheat are LD plants, flowering earlier under LDs than SDs (photoperiod responsive or photoperiod sensitive). However, both species have day-neutral (or photoperiod insensitive) mutations that enable rapid flowering under SD or LD conditions. The control of *Vrn*-*2* expression by photoperiod and the availability of different day-neutral mutations provided a novel opportunity to investigate the interaction between photoperiod and vernalization. In barley we used an *early maturity8* (*eam8*) mutation that affects an orthologue of the Arabidopsis circadian clock gene *EARLY FLOWERING3* (*ELF3*) gene (Faure et al. 2012; Zakhrabekova et al. 2012). *eam8* affects the expression of genes that are components of the circadian clock (*HvCCA1*, *HvTOC1* and *HvGI*) and causes constitutive activation of photoperiod response with high levels of *Ppd*-*H1* and *HvFT1* expression. This leads to rapid flowering under SD or LD conditions (Faure et al. 2012). *eam8* mutant stocks are in spring barley varieties that do not require vernalization because of mutations in *Vrn*-*1* and *Vrn*-*2*. For our experiments we crossed *eam8* into a winter barley background with functional *Vrn*-*1* and *Vrn*-*2* alleles.

In wheat we used the *Ppd*-*D1a* mutation. This has a promoter deletion associated with constitutive expression. In contrast to *eam8* the *Ppd*-*D1a* mutation does not affect the expression of genes in the circadian clock (*TaCCA1*, *TaTOC1* or *TaGI*), but does affect downstream photoperiod pathway components resulting in induction of *TaFT1* expression under SD conditions (Beales et al. 2007; Wilhelm et al. 2009; Shaw et al. 2012). We also utilised the observation that SDs can partially substitute for cold treatment in some wheat varieties, a phenomenon known as “short-day vernalization” (Purvis and Gregory 1937; Roberts et al. 1988) or “short-day induction” (Evans 1987). We investigated *Vrn*-*2* expression in wild-type, *eam8* and *Ppd*-*D1a* mutant genotypes and related this to flowering time in vernalized (V) and UV plants. This enabled us to study the interaction between photoperiod and vernalization pathways and to determine whether variation in short-day vernalization behaviour was attributable to allelic variation at *Ppd*-*1*.

## Materials and methods

### Plant material

Development of the ‘Igri (*eam8*)’ lines is described in Faure et al. (2012). Seeds of wheat varieties were obtained from the Genetic Resources Unit of the John Innes Centre.

### Growth conditions for barley

Seeds were germinated to coleoptile lengths of 1–3 cm and V for 6 weeks at 4 °C in 8 h days. Ten days before the end of vernalization additional seeds were germinated and V and UV plants were grown together in SD (9 h light, 15 h dark) or LD (16 h light, 8 h dark) conditions (136 μmol m^−2^ s^−1^ light; 16 °C). Ten plants of each genotype were grown for each treatment combination. Flowering time was recorded as the date when awns had emerged 2 cm from the flag leaf.

For quantitative RT-PCR plants were grown as above and entire above-ground material from seedlings were sampled into liquid nitrogen on the days and at the time points described in “Results”. Each sample comprised material from three seedlings, and three samples were taken per treatment. Error bars on graphs show the standard error of the mean (*n* = 3).

For the continuous light experiment UV ‘Igri’ seedlings were grown in a 12 h light/12 h dark cycle (136 μmol m^−2^ s^−1^ light; 16 °C) for 16 days and then given continuous light for the remainder of the experiment. Three samples of above-ground material (biological replicates) were taken into liquid nitrogen every 4 h with each sample comprising material from three seedlings.

### Growth conditions for wheat

Seeds were germinated to coleoptile lengths of 1–3 cm and grown for 4 or 8 weeks in SD (9 h light, 15 h dark; 136 μmol m^−2^ s^−1^ light; 16 °C). Leaf tissue (20 mm^2^) of each individual was taken after 10 days for DNA extraction and *PpdD1* allele typing as in Beales et al. (2007).

For quantitative PCR, at 19 days post-germination four individuals from each parental line (‘Maris Templar’, ‘Cappelle-Desprez’ and ‘Krasnodar39’) and four individuals from each category (parental homozygous and heterozygous) from the ‘Cappelle-Desprez’ × ‘Krasnodar39’ cross were selected at random and assayed for *Vrn*-*2* expression were sampled for genotyping and *Vrn*-*2* expression. The results presented in Fig. 6 are therefore the mean and standard deviation of four observations per sample. After the SD treatment, the plants were moved to a glasshouse with natural LDs (>14 h light) and flowering time was recorded as the date of ear emergence (when the spike was 50 % emerged from the flag leaf).

### Quantitative RT-PCR

RNA was extracted, cDNA was synthesised and samples were processed essentially as described in Shaw et al. (2012). Tri-Reagent (Sigma) was used for RNA extraction and MMLV-RTase (Invitrogen) with Oligo-dT (12–18) for reverse-transcription. Quantification was achieved using either an Opticon Real-Time PCR instrument (Bio-Rad) or a Roche LightCycler 480 instrument. SybrGreen Fluorescence chemistry was used to quantify amplification. Cycle threshold values were converted into relative mRNA abundance using the Δ*C*_t_ method using the reaction efficiency values calculated by the instrumentation software. The abundance calculations were performed as $E^{{ - \Updelta C_{\text{t}} }}$ where *E* is the reaction efficiency and then normalised by division with the normalising factor, calculated in the same way either singly (18s rRNA, wheat experiments and barley entrainment experiment) or as a geometric mean (18s rRNA and actin mRNA, barley 24 h experiments; Vandesompele et al. 2002) to control for fluctuations in cDNA synthesis efficiency. PCR primers used are listed in Online Resource 1.

## Results

### Phenotypes of barley *eam8* mutant plants

Development of *eam8* lines is described in Faure et al. (2012), but briefly the *eam8* (*ea8.k* allele) mutation was crossed to the winter barley ‘Igri’ after which self-pollinated plants were V and selected for early flowering in SDs. From these, plants were selected that were homozygous for the ‘Igri’ alleles at *Vrn1* and *Vrn2* (determining vernalization requirement) and the photoperiod-sensitive *Ppd*-*H1* ‘Igri’ allele using a combination of PCR-based markers (Turner et al. 2005; Yan et al. 2004; Fu et al. 2005) and phenotype screens. These plants are referred to as ‘Igri(*eam8*)’.

**Fig. 1:**
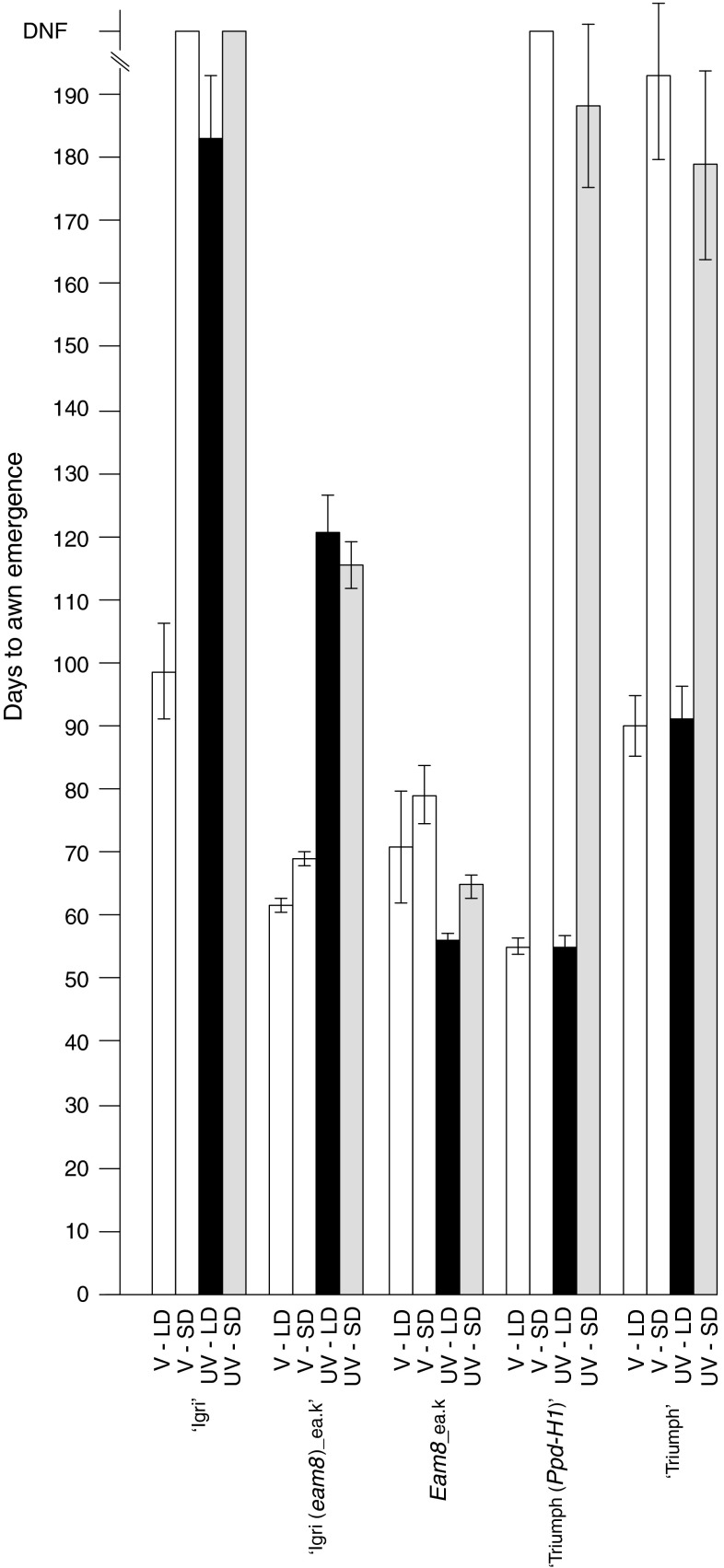
Days to awn emergence of barley cultivars and introgression lines. Vernalized (V) and unvernalized (UV) plants were grown under short days (SD) and long days (LD). *Error bars* indicate standard error of the mean. Flowering times of vernalized plants of ‘Igri’, ‘Igri(*eam8*)’ and the *eam8* parent were previously published in Faure et al. (2012)

Flowering time (days to awn emergence on the main stem) was recorded for V and UV plants in controlled environment cabinets with SD (9 h light) or LD (LD; 16 h light) conditions (Fig. 1). For the purposes of this report the term “unvernalized” (UV) refers to plants which have not been subjected to vernalization treatment.

The parental lines behaved as expected from previous studies and their known *Ppd* and *Vrn* genotypes. Flowering in ‘Igri’ (winter barley parent) was delayed by at least 80 days in UV plants (LDs or SDs) and in V plants in SDs. The *eam8* parent was a spring barley and flowered at similar times in SDs and LDs with or without vernalization, as expected. Additional controls were ‘Triumph’, which carries the *ppd*-*H1* mutation, and ‘Triumph(*Ppd*-*H1*)’ which is an introgression line carrying the active *Ppd*-*H1* allele from ‘Igri’ (Turner et al. 2005). ‘Triumph’ (spring growth habit; *VrnA1c*; Casas et al. 2008) flowered earlier in LDs than SDs and this difference was enhanced by the *Ppd*-*H1* allele. Neither genotype was affected by vernalization, consistent with previous results.

The flowering repressor *Vrn*-*2* is expressed under LDs but not SDs in wild-type plants (Dubcovsky et al. 2006; Trevaskis et al. 2006). This suggested that UV ‘Igri(*eam8*)’plants would be early flowering under SDs (where VRN-2 is predicted to be absent), and late flowering under LDs (where VRN-2 is normally expressed). However, UV ‘Igri(*eam8*)’ plants flowered at the same time in SD or LD conditions and were approximately 50 days later than V plants (Fig. 1). This suggested that *Vrn*-*2* was expressed under SD or LD conditions in UV *eam8* plants to partially suppress the early flowering phenotype.

### Expression of *Vrn*-*2* in wild type and *eam8* mutant barley plants

**Fig. 2:**
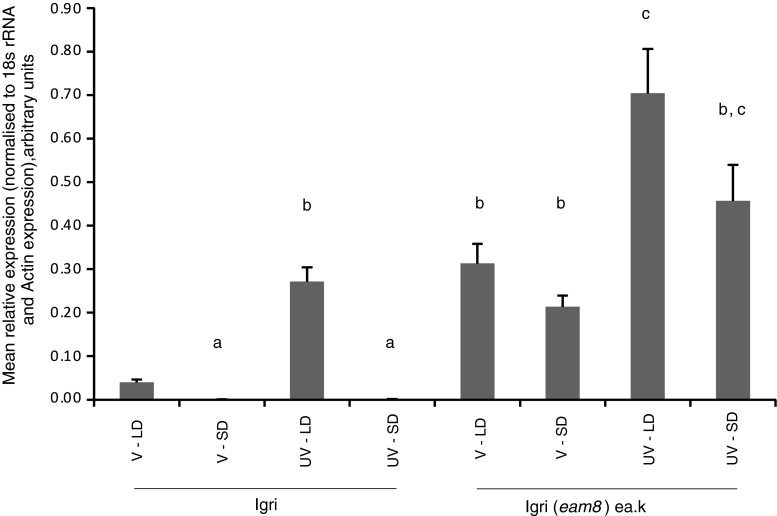
*Vrn*-*2* expression 8 h after dawn in ‘Igri’ and ‘Igri(*eam8’*) plants comparing vernalized (V) or unvernalized (UV) plants grown under short days (SD) or long days (LD). *Error bars* indicate standard error of the mean, and columns bearing the *same lettering* are not significantly different from each other at the 95 % confidence level (one-way ANOVA)

In UV ‘Igri’ plants *Vrn*-*2* was expressed under LDs but was practically undetectable under SDs, consistent with previous studies (Dubcovsky et al. 2006; Trevaskis et al. 2006). *Vrn*-*2* was expressed in UV ‘Igri(*eam8*)’ plants under SD or LD conditions. Expression was significantly higher than wild type under SDs (Fig. 2). Previous results from a microarray experiment showed that the *eam* mutant constitutively behaved as if under LDs (Faure et al. 2012). Figure 2 shows that this behaviour extends to the expression of *Vrn*-*2*, which was not represented on the array. Expression was reduced after vernalization in ‘Igri’ plants as expected. A fall in the average level was also seen in ‘Igri(*eam8*)’ plants, especially under LDs, showing that *eam8* still responds to vernalisation.

**Fig. 3:**
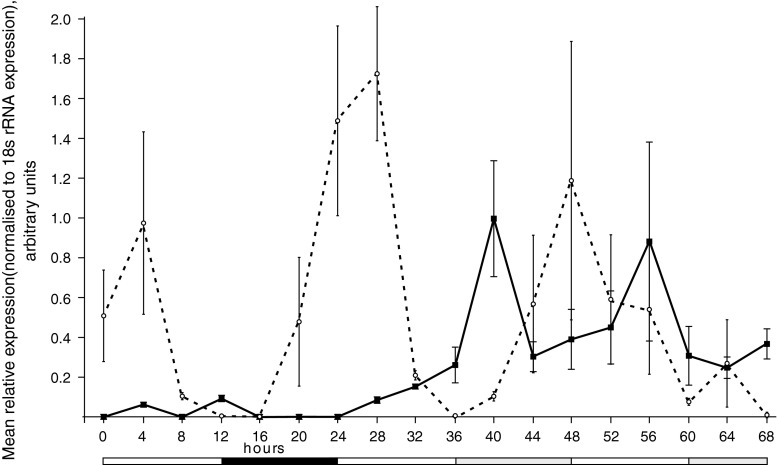
*Vrn*-*2* (*solid line*) and *HvCCA1* (*dotted line*) expression time courses in unvernalized ‘Igri’ plants entrained under 12 h light/12 h dark cycles then subjected to constant light. *Error bars* indicate standard error of the mean

*Vrn*-*2* is a member of the CCT domain family of genes. Other members of this family are known to be components of, or regulated by, the circadian clock (Mizuno and Nakamichi 2005). *Vrn*-*2* has previously been shown to have a diurnal change in expression when measured together with a closely related CCT gene (*HvZCCTa* and *HvZCCTb* in Trevaskis et al. 2006). These data suggested that *Vrn*-*2* might have circadian control in wild-type plants. To test this we entrained UV ‘Igri’ plants in 12 h light/12 h dark conditions for 16 days and then moved them to continuous light and measured expression at 3 h intervals over 48 h. *Vrn*-*2* expression was very low in the 12 h/12 h conditions, but rose rapidly 16 h after transfer to continuous light. Expression then fell during the subjective night and rose again at 56 h (Fig. 3). This showed circadian control of *Vrn*-*2* in wild-type plants and also showed that expression was strongly induced by the first exposure to 16 h or more of light. The plants sampled for expression in Fig. 2 were harvested at the start of the day which explains the relatively low level of *Vrn*-*2* expression in wild-type plants.

### Expression of *Ppd*-*H1*, *HvCO1,HvFT1* and *Vrn*-*1* in wild type and *eam8* mutant barley plants

**Fig. 4:**
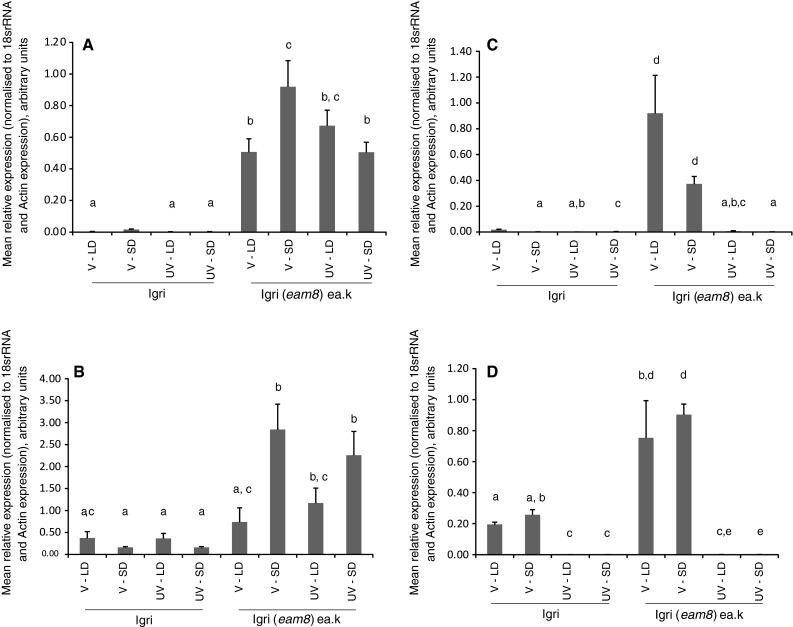
Expression of photoperiod pathway genes at dawn in ‘Igri’ and ‘Igri(*eam8*)’ plants comparing vernalized (V) or unvernalized (UV) grown under short days (SD) or long days (LD). *Error bars* indicate standard error of the mean, and columns bearing the *same lettering* are not significantly different from each other at the 95 % confidence level (one-way ANOVA). **a** *Ppd*-*H1,* **b** *HvCO1*, **c** *HvFT1*, **d** *Vrn*-*1*

To investigate the effect of vernalization further, expression of *Ppd*-*H1*, *HvCO1*, *HvFT1* and *Vrn*-*1* were compared in V and UV seedlings of ‘Igri’ and ‘Igri(*eam8*)’ grown under SD and LD conditions and sampled at subjective dawn (Fig. 4). This time point was selected because expression levels were previously found to be low in ‘Igri’ and high in ‘Igri(*eam8*)’ using V plants under SDs (Faure et al. 2012). Plants in the present study behaved similarly. *PpdH1* expression was almost undetectable in ‘Igri’ wild-type plants, but was expressed under all conditions in ‘Igri(*eam8*)’ plants (Fig. 4a). Neither changes in day length or vernalization caused consistent changes in *PpdH1* expression in ‘Igri(eam8)’ plants. Greater expression was seen in V plants in SD than LD, and vice versa for UV plants; vernalization produced an increase in expression in SD plants but a non-significant reduction in LD plants. There was a tendency for *HvCO1* expression to be increased in SD relative to LD for ‘Igri(*eam8*)’ plants (Fig. 4b), while the reverse was seen in ‘Igri’ plants, but vernalization had no effect on *HvCO1* expression, which was always low in ‘Igri’ and high in ‘Igri(*eam8*)’. In contrast, vernalization has a strong effect on *HvFT1* expression (Fig. 4c), which was greatly reduced in UV plants of both genotypes, most likely due to high levels of *Vrn*-*2*. The deregulation of expression of *Vrn*-*2* seen in ‘Igri(*eam8*)’ plants was not observed for *Vrn*-*1* (Fig. 4d), where expression was almost undetectable in UV plants whether or not the *eam8* allele was present. In V plants the *eam8* allele was associated with increased expression of *Vrn*-*1*.

These results show that *eam8* plants lost the normal day length control of *Vrn*-*2*, expressing the gene under SD and LD conditions (Fig. 2), consistent with the delayed flowering phenotype under both conditions. One explanation is that *Vrn*-*2* is directly controlled by the circadian clock which is disrupted in the *eam8* mutant (Faure et al. 2012). An alternative possibility is that *Vrn*-*2* is controlled by genes regulated by the clock but downstream of the clock itself. For example, *Ppd*-*H1* expression is deregulated and increased in *eam8* plants. To test the control of *Vrn*-*2* further, we examined wheat plants carrying the dominant *Ppd*-*D1a* mutation. This causes over expression of *Ppd*-*D1* and a day-neutral early flowering phenotype, but does not affect the expression of the circadian clock genes *TaCCA1*, *TaTOC1* or *TaGI* (Shaw et al. 2012).

### Expression of *Vrn*-*2* in wild type and photoperiod-insensitive (*Ppd)* mutant wheat plants

**Fig. 5:**
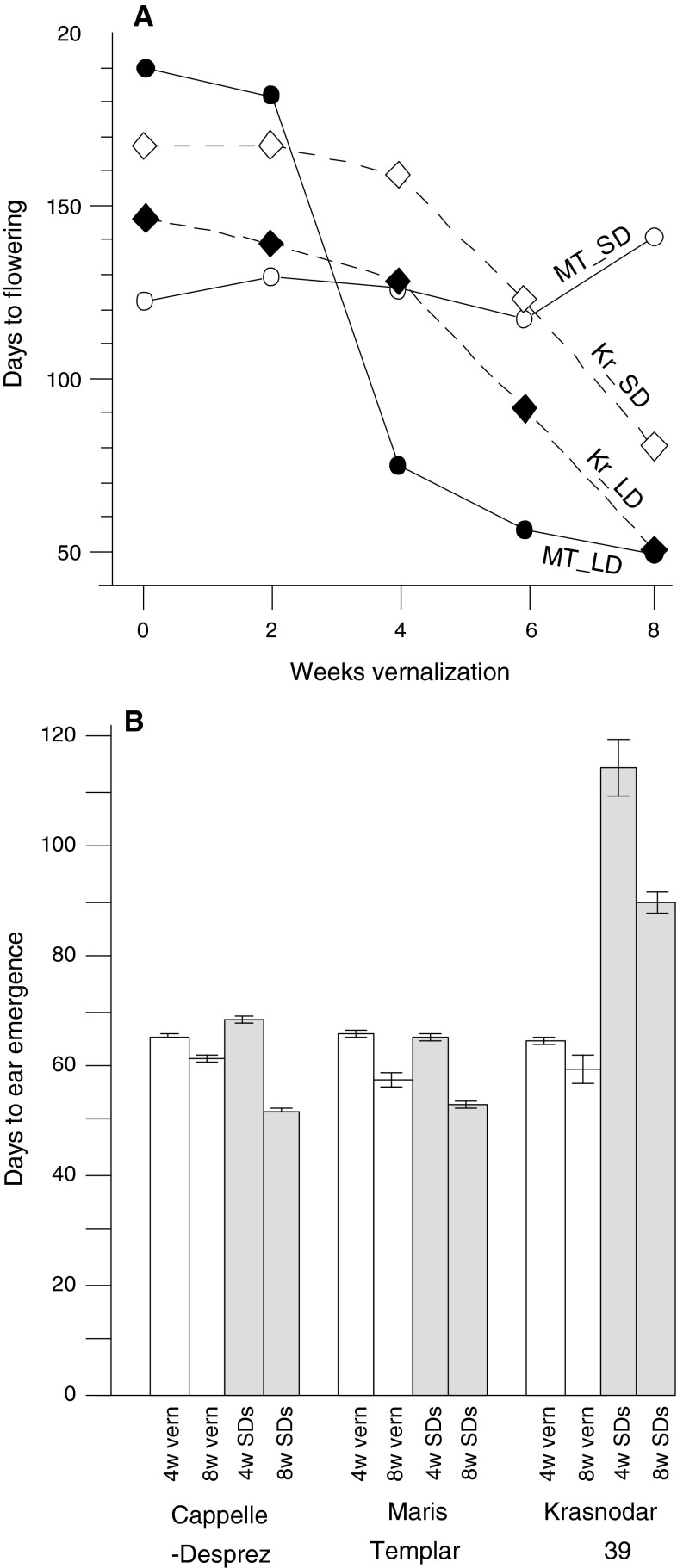
Days to flowering in wheat varieties differing in short-day vernalization response. **a** Days to flowering of wheat cultivars ‘Maris Templar’ (MT) and ‘Krasnodar 39’ (Kr) subjected to different vernalization periods then grown under continuous short days (SD) or long days (LD). Redrawn from Figure 3 of Davidson et al. (1985). **b** Days to ear emergence of wheat cultivars ‘Maris Templar’, ‘Cappelle-Desprez’ and ‘Krasnodar 39’ after 4 or 8 weeks vernalization (‘vern’) or an equivalent period in short days (‘SD’) and a non-vernalizing temperature of 16 °C before transfer to a glasshouse under natural warm LD conditions

To study *Vrn*-*2* expression in wheat, we utilised a known observation that wheat varieties differ in their interaction between day length and vernalization. Some winter (vernalization requiring) varieties of cereals are able to flower after a period of growth in SDs at non-vernalizing temperatures, a phenomenon known as “short-day vernalization” (Purvis and Gregory 1937; Roberts et al. 1988). In diploid wheat (*T. monococcum*) this has been shown to be associated with loss of *Vrn*-*2* expression (Dubcovsky et al. 2006). It had also been noted that varieties which failed to respond to short-day vernalization were often known to carry the photoperiod-insensitive (day neutral) mutation *Ppd1*. For this paper we reinvestigated hexaploid winter wheat varieties previously studied by Davidson et al. (1985). In Davidson et al.’s experiments different lengths of vernalization treatment were followed by growth at non-vernalizing temperatures in SD or LD conditions. They showed that the English variety ‘Maris Templar’ flowered after approximately 125 days in SDs irrespective of the vernalization treatment while in LDs flowering was strongly delayed in plants given less than 4 weeks vernalization and occurred most rapidly in plants that were fully V (Fig. 5a). Thus, ‘Maris Templar’ exhibited “short-day vernalization”. In contrast, the Russian variety ‘Krasnodar 39’ did not, and behaved similarly when grown under SD or LD conditions, with flowering always delayed by no or shorter vernalization times (Fig. 5a).

We used a different protocol to confirm the observation that SDs could substitute for vernalization. We grew plants of ‘Maris Templar’, ‘Cappelle-Desprez’ (also studied by Davidson et al. (1985) and showed to behave like ‘Maris Templar’) and ‘Krasnodar 39’ for 0, 4 or 8 weeks at a non-vernalizing temperature of 16 °C in SDs (9 h light) before transferring plants to a glasshouse under natural warm LD conditions. Flowering times (days to ear emergence after transfer to the glasshouse; Fig. 5b) showed that the SD treatment could substitute for equivalent periods of vernalization in ‘Maris Templar’ and ‘Cappelle-Desprez’ in that plants reached ear emergence at equivalent dates (N.B. at transfer the SD grown plants were considerably larger and more developed). The 4 or 8 week SD treatment was much less effective in ‘Krasnodar 39’ (Fig. 5b), consistent with the inability of this variety to respond to short-day vernalization found by Davidson et al. (1985).

**Fig. 6:**
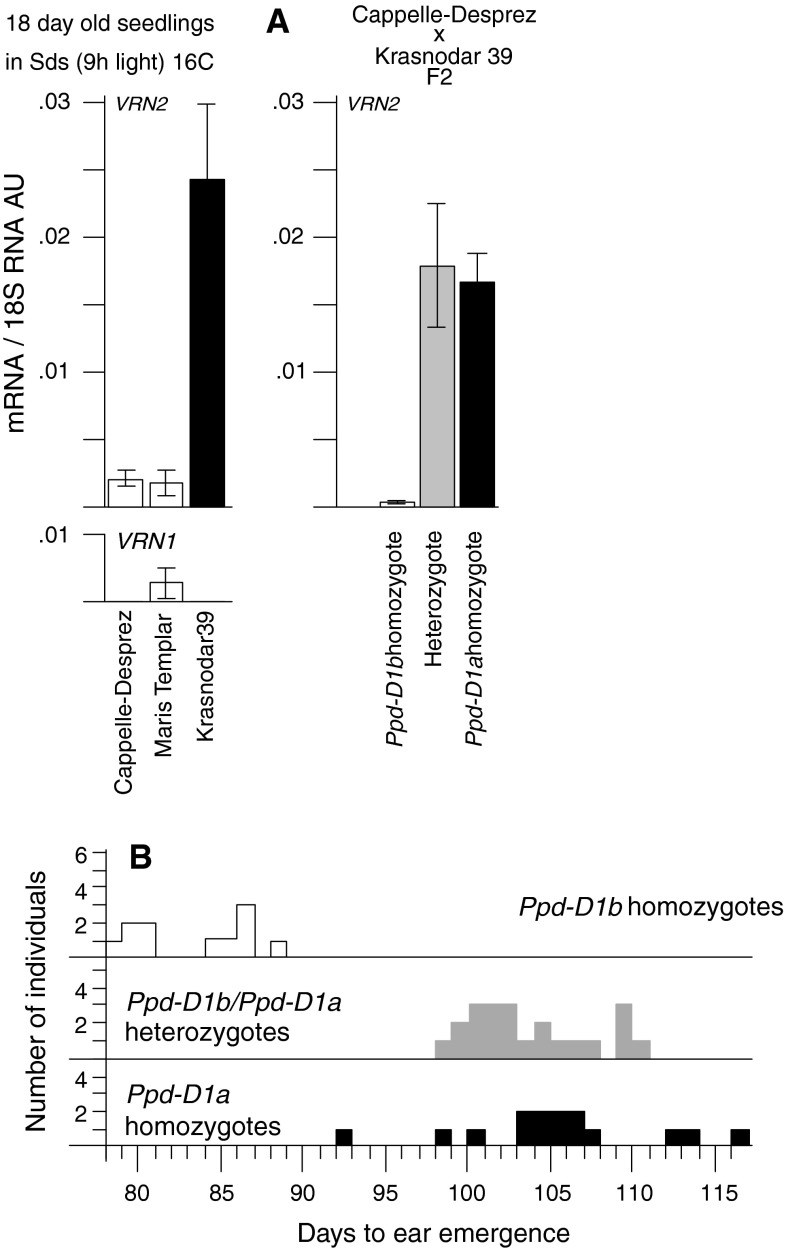
*Vrn*-*2* expression and flowering times in wheat. **a** *Vrn*-*1* and *Vrn*-*2* expression in wheat seedlings grown for 18 days in SD. *Left panel* shows expression in seedlings of cultivars ‘Maris Templar’, ‘Cappelle-Desprez’ and ‘Krasnodar 39’, and *right panel* shows expression in individuals of a ‘Cappelle-Desprez’ × ‘Krasnodar 39’ F_2_ population when classified according to *Ppd*-*D1* allele status. *Error bars* indicate standard error of the mean. **b** Days to ear emergence of a ‘Cappelle-Desprez’ × ‘Krasnodar 39’ F_2_ population subjected to 4 weeks in SD then moved to natural LD. Individuals are classified according to *Ppd*-*D1* allele status. *Open squares* are *PpdD1b* individuals, *black squares* are *PpdD1a* individuals, and *grey squares* are heterozygotes

Genotyping using assays described in Beales et al. (2007) showed that ‘Maris Templar’ and ‘Cappelle-Desprez’ were photoperiod-sensitive varieties (wild-type *Ppd*-*1* alleles) while ‘Krasnodar 39’ carried the photoperiod-insensitive (day neutral) *Ppd*-*D1a* mutation. To determine if this explained the difference in short-day vernalization response, we first assayed *Vrn*-*2* expression in UV plants. Whilst all three varieties showed similar levels of *Vrn*-*2* expression under LD (data not shown), only ‘Krasnodar 39’ expressed *Vrn*-*2* strongly under SD conditions (Fig. 6a).

We then examined the relationship between *Ppd*-*D1*, SD expression of *Vrn*-*2*, and SD-vernalization competence in a segregating population. F_2_ seedlings from a ‘Cappelle-Desprez’ × ‘Krasnodar 39’ cross were grown initially at 16 °C in SDs (9 h light). Plants were genotyped for *Ppd*-*D1* and at 19 days post-germination four individuals from each category (parental homozygous and heterozygous) were selected at random and assayed for *Vrn*-*2* expression. Plants homozygous for the wild-type (photoperiod sensitive) *Ppd*-*D1b* allele had very low levels of *Vrn*-*2* expression while expression was high in heterozygotes and in plants homozygous for the dominant day-neutral *Ppd*-*D1a* allele (Fig. 6b). After 4 weeks the plants were moved to natural LDs in a glasshouse and days to ear emergence were recorded. Photoperiod-sensitive *Ppd*-*D1b* homozygote individuals flowered ahead of plants carrying the mutant *Ppd*-*D1a* allele (Fig. 6c), showing that variation in SD-vernalization in these genotypes could be explained by allelic variation at *Ppd*-*1* and associated variation in *Vrn*-*2* expression. *Ppd*-*D1a*, like *eam8*, therefore causes plants to behave as if under LDs in terms of photoperiod response and *Vrn*-*2* expression.

## Discussion

The flowering repressor *Vrn*-*2* was previously shown to be expressed under LD but not SD conditions in wild-type plants (Dubcovsky et al. 2006; Trevaskis et al. 2006). It was also shown to have diurnal variation in expression in LDs (Trevaskis et al. 2006). In this paper, we show that *Vrn*-*2* is regulated by the circadian clock. Use of two contrasting mutations that cause early flowering under SD or LD allowed the regulation of *Vrn*-*2* and the interaction of photoperiod and vernalization to be explored in more detail.

In terms of photoperiod response the day-neutral *eam8* mutation of barley and the *Ppd*-*D1a* mutation of wheat cause the plants to behave as if they are under LDs, irrespective of the actual day length. A result of this is that plants with either mutation express *Vrn*-*2* under SD or LD conditions. *ELF3* is an important component of the circadian clock in Arabidopsis (McWatters et al. 2000) with *elf3* mutants showing clock arrhythmia under constant conditions (Hicks et al. 2001; Thines and Harmon 2010). *EAM8* is a barley orthologue of *ELF3* and the early flowering *eam8* phenotype results from loss of gene function (Faure et al. 2012; Zakhrabekova et al. 2012). *eam8* mutants have altered expression of circadian clock genes (*HvCCA1*, *HvTOC1* and *HvGI*) and downstream components of the photoperiod pathway so that *Ppd*-*H1* expression is increased and *HvFT1* is expressed under SD or LD conditions (Faure et al. 2012). The *Ppd*-*D1a* mutant of wheat has no effect on the circadian clock, at least in normal light/dark cycles, but causes constitutive expression of the *Ppd*-*D1* gene itself and expression of *TaFT1* under SDs (Shaw et al. 2012).

Our data show there are at least two routes to *Vrn*-*2* expression in SD: via clock disruption and associated activation of the photoperiod pathway, as in ‘Igri(*eam8*)’; and by activation of the photoperiod pathway without clock disruption using the *PpdD1a* allele. From this we conclude that *Vrn*-*2* is not directly regulated by the clock, but is instead under the control of one or more clock-regulated downstream components. Although *Ppd*-*1* itself is a candidate it should be noted that *Ppd*-*1* is expressed under SDs in wild-type plants even though *Vrn*-*2* is not. Therefore, it would be the altered timing of *Ppd*-*1* expression during the day that is significant in the mutants. This model predicts that a reduction in *Ppd*-*H1* activity would reduce *Vrn*-*2* expression. The recessive *ppd*-*H1* mutation in barley provides an appropriate test as this is a loss or reduced function allele that delays flowering in LDs but has no effect in SDs (Turner et al. 2005). However, Hemming et al. (2008) found that *Vrn*-*2* levels in LDs did not differ between *Ppd*-*H1* and *ppd*-*H1* plants. Therefore, the mechanism by which the day-neutral mutations affect *Vrn*-*2* expression remains unclear and requires further study.

It has been proposed that *Vrn*-*2* affects flowering by repressing *FT1* (*Vrn*-*3*) expression (Hemming et al. 2008; Chen and Dubcovsky 2012). The observation in this paper that vernalization did not repress two candidate upstream regulators of *FT1* (*Ppd*-*H1* and *HvCO1*) is consistent with this, supporting that idea that vernalization and photoperiod pathways converge on *FT* and its orthologues in Arabidopsis and cereals, despite the fact that different vernalization genes are involved (*FLC* in Arabidopsis and *Vrn*-*1*/*Vrn*-*2* in cereals). From these results we explain the observed effects of vernalization in this paper as follows. In barley, flowering in UV ‘Igri(*eam8*)’ plants under SDs or LDs was repressed because *Vrn*-*2* was expressed under both conditions (Fig. 2). Flowering occurred significantly earlier than in the UV ‘Igri’ parent, suggesting a quantitative competition between the promoting effect of the photoperiod pathway, which is strongly enhanced in *eam8* plants, and the repressive effect of the vernalization pathway. This conclusion is also supported by the genetic interactions between *Vrn*-*1*, *Vrn*-*2* and *Ppd*-*H1* described by Hemming et al. (2008). In wheat we interpret flowering times and the phenomenon of “short-day vernalization” in Fig. 5a as follows: in photoperiod-sensitive winter wheat varieties such as ‘Maris Templar’ and ‘Cappelle-Desprez’ the flowering time in SDs is consistent irrespective of the vernalization treatment because there is no flowering repression (*Vrn*-*2* is not expressed in SDs) and no photoperiodic promotion. The fact that flowering occurs in SDs implies the existence of an additional pathway which could involve ambient temperature or age. *FT3* (related to *FT1*) may be significant as in barley it is gradually upregulated in SDs (Faure et al. 2007). It seems likely that HvFT3, like HvFT1, is involved in the integration of signals from the vernalization and photoperiod pathways, as its expression is affected by both photoperiod (Faure et al. 2007) and by the requirement for a functional copy of Vrn-2. Casao et al. (2011) found that barley plants with the spring growth habit caused by Vrn-2 deletions expressed HvFT3 at high levels in the absence of vernalization, and that this deregulation allowed expression in normally repressive LD conditions, leading them to propose Vrn-2 as a repressor of HvFT3.

In LDs there is high *Vrn*-*2* expression in plants that are not V, and this suppresses flowering. As vernalization proceeds, *Vrn*-*2* gradually becomes repressed and the LD photoperiod response is increasingly able to promote flowering. In ‘Krasnodar 39’ the *Ppd*-*D1a* mutation causes the plants to behave as if they are under LDs, irrespective of the actual day length, so that *Vrn*-*2* is expressed under SD or LD conditions. Consequently, SD and LD flowering times were similar for each vernalization time (Fig. 5a). Flowering in LD was always slightly earlier than in SD, possibly because of an additional promoting effect from the wild-type *Ppd* alleles on the other genomes. In our experiments (Fig. 5b), growth in SDs for 4 or 8 weeks was sufficient to commit wild-type plants to flowering, again because there was no *Vrn*-*2* mediated repression. SDs were less efficient for ‘Krasnodar 39’ because *Vrn*-*2* was expressed due to the presence of the day-neutral *Ppd*-*D1a* mutation.

Work in rice has shown that the *Ghd7* gene, which is a repressor of flowering in LDs, is a member of the same grass-specific subgroup of CCT domain genes as *Vrn*-*2* (Xue et al. 2008; reviewed by Higgins et al. 2010). This suggests that LD suppression was an ancestral property of cereals and that the evolution of temperate cereals involved at least two key changes. One was a change to LD promotion for the photoperiod pathway. The second was the evolution of a vernalization pathway involving *Vrn*-*1* being able to repress *Vrn*-*2* after a period of low temperature. These changes enabled ancestral cereals to adopt a winter annual life style with suppression of flowering in late summer and autumn and rapid flowering under increasing day lengths in the following spring.

## Electronic supplementary material

Below is the link to the electronic supplementary material.

Supplementary material 1 (DOCX 16 kb)
