# Supplementary material for: The effect of day-neutral mutations in barley and wheat on the interaction between photoperiod and vernalization
Source: Theor Appl Genet. 2013 Jun 5;126(9):2267–77. doi: 10.1007/s00122-013-2133-6 (PMC3755224; doi:10.1007/s00122-013-2133-6)
Supplement: Supplementary file 1 — Supplementary material 1 (DOCX 16 kb) [file 122_2013_2133_MOESM1_ESM.docx]

Online Resource 1: Theoretical & Applied Genetics

The effect of day neutral mutations in barley and wheat on the interaction between photoperiod and vernalization.

Adrian S Turner^1^, Sébastien Faure^3^, Yang Zhang^2^ and David A Laurie^1^

^1^Crop Genetics Department and ^2^Department of Metabolic Biology, John Innes Centre, Colney Lane, Norwich, NR47UH, UK.

^3^Present address: Biogemma, Cereals Genetics and Genomics, 63028 Clermont Ferrand cedex 2, France

Corresponding author: Adrian Turner

e-mail: [adrian.turner-crls@jic.ac.uk](mailto:adrian.turner-crls@jic.ac.uk)

| Assay | Primers | Reference |
| --- | --- | --- |
| 18s rRNA | atacgtgcaacaaaccc  ctacctccccgtgtca | Turner *et al*., 2005 |
| Hv Actin | gccgtgctttccctctatg  gcttctccttgatgtccctta | Trevaskis *et al.,* 2006 |
| *HvCO1* | ggggcagagcaggctgcctc  tggcttctctctccttggagc | Turner *et al*., 2005 |
| *Ppd-H1* | ttgagctgagcctgaagag  tatagctaggtgcgtggcg | Turner et al., 2005 |
| *HvFT1* | ccaaccttagagagtatctccact  ccctggtgttgaagttctgg | Turner et al., 2005 |
| *Vrn-H1* | tgaagctcagaaatggattcg  tatgagcgctactcttatgc | Trevaskis *et al*., 2006 |
| *Vrn-H2* | gagccaccatcgtgccattc  gccgcttcttcctcttctc | Trevaskis *et al*., 2006 |
| *TaVrn2* | atcaccttcgctgctctctc  cccacatcgtgccattttac | Distelfeld *et al*., 2009b |
